# Assessing Chemical Intolerance in Parents Predicts the Risk of Autism and ADHD in Their Children

**DOI:** 10.3390/jox14010022

**Published:** 2024-03-05

**Authors:** Raymond F. Palmer, David Kattari, Rodolfo Rincon, Claudia S. Miller

**Affiliations:** 1 Department of Family and Community Medicine, University of Texas Health Science Center at San Antonio, San Antonio, TX 78229, USA; rorin042059@gmail.com (R.R.); millercs@uthscsa.edu (C.S.M.); 2 Marilyn Brachman Hoffman Foundation, Fort Worth, TX 75202, USA; dkattari13@gmail.com

**Keywords:** environment, exposure, toxicants, autism, Asperger’s, QEESI, TILT, mast cells, fossil fuels, xenobiotics

## Abstract

Background: We sought to replicate our 2015 findings linking chemical intolerance in parents with the risk of their children developing autism and/or ADHD. Drawing upon our 2021 discovery of a strong association between chemical intolerance and mast cells, we propose an explanation for this link. Methods: In a population-based survey of U.S. adults, we used the internationally validated Quick Environmental Exposure and Sensitivity Inventory (QEESI) to assess symptom severity and chemical intolerance. Parents were asked how many of their biological children had been diagnosed with autism and/or ADHD. Results: Parents with chemical intolerance scores in the top versus bottom tenth percentile had 5.7 times the risk of reporting a child with autism and 2.1 times for ADHD. Conclusions: High chemical intolerance scores among parents of children with autism, coupled with our 2021 discovery of mast cell activation as a plausible biomechanism for chemical intolerance, suggest that (1) the QEESI can identify individuals at increased risk, (2) environmental counseling may reduce personal exposures and risk, and (3) the global rise in autism and ADHD may be due to fossil-fuel-derived and biogenic toxicants epigenetically “turning on” or “turning off” critical mast cell genes that can be transmitted transgenerationally. It is important to note that this study was observational in nature; as such, further research is needed using controlled trials to confirm causality and explore the proposed mechanism.

## 1. Introduction

### 1.1. Autism

Autism is a behaviorally defined neurodevelopmental disorder characterized by deficits in language, communication, and social function [1]. The most recent prevalence estimates range from 1 in 30–44 U.S. births, with an estimated global prevalence of 1 per 100 children [2,3,4]. Autism prevalence in the U.S. has increased by 6–15% each year from 2002 to 2020, with a recent sharp increase in autism prevalence among Black (2.9%), Hispanic (3.2%), and Asian or Pacific Islander (3.3%) children [2]. A portion of the recent exponential rise in autism overall has been attributed to increased awareness and screening, better access to healthcare, broadened diagnostic criteria, and/or better diagnostic practices [4]. This may be especially true for minorities; however, these assertions have not so far been substantiated [5].

The interface between the emerging genomic and exposomic sciences presents various methodological challenges for researchers in terms of understanding the complex interactions between an individual’s biology and multiple environmental exposures [6]. Currently, Gene/Environment (GxE) interactions are widely regarded as the most probable explanation for most autism cases, especially given the fact that genes are selectively targeted by diverse xenobiotics [6,7,8,9]. These observations suggest the need for improved genetic screening and public health strategies in order to reduce toxic exposures.

### 1.2. Attention Deficit Hyperactivity Disorder (ADHD)

Attention Deficit Hyperactivity Disorder (ADHD) is diagnosed by a clinical interview during which various criteria are assessed, including difficulty paying attention, poor impulse control, and overactivity. Like autism, ADHD is more common in males. Worldwide, ADHD affects approximately 6% of youth and 2.5% of adults [10]. There is substantial overlap between autism and ADHD, with their co-occurrence estimated to be between 28% and 78% [11]. Other comorbidities include obesity, asthma, allergies, diabetes mellitus, and immune and metabolic disorders [12,13,14,15,16,17,18].

Similar to autism, ADHD is rarely caused by a single gene or environmental agent but is thought to result from the combined effects of various genetic and environmental factors [19]. Environmental factors, including heavy metals, organophosphate pesticides, cigarette smoke, and phthalates are associated with, and increase the risk of, ADHD [20,21,22,23]. Synthetic food dyes and lower levels of omega-3 polyunsaturated fatty acids are also implicated [24,25]. Interestingly, low maternal vitamin D levels also increase the risk for ADHD and autism [26,27]. The shared risk factors and comorbidities between autism and ADHD suggest potentially valuable directions for future research in both humans and animal models [28,29,30].

### 1.3. Autism, ADHD, and Chemical Intolerance (CI)

In 2015, we reported that mothers who suffer from chemical intolerance have three times the odds of reporting a child diagnosed with autism and 2.3 times the odds of reporting a child diagnosed with ADHD compared to control mothers [31]. Here, we examine the association between autism, ADHD, and Toxicant-Induced Loss of Tolerance (TILT). When TILT was first described in 1996, there was no known biomechanism to explain worldwide observations of individuals developing chemical, food, and drug intolerances following exposures to toxicants. It was not until a quarter-century later, in 2021, that we proposed and published mast cell alteration by toxic exposures as the underlying biomechanism for chemical intolerance and TILT [32,33,34].

CI is characterized by multisystem symptoms and intolerances for chemical inhalants, foods/food additives, and drugs [32]. Any and all organ systems can be involved [35,36]. Prevalence estimates vary according to whether CI is clinically diagnosed (0.5–6.5%) or self-reported (average ~20%) [37,38,39,40,41]. Researchers in the U.S. and Japan have noted increased CI prevalence over a 10-year period [42,43].

In prior papers, we have described how CI begins with a single, high-level exposure to a toxicant such as a pesticide, chemical release, or repeated or chronic lower-level exposures to toxicants such as volatile organic compounds (VOCs) in a “sick” building [32,34]. For decades, researchers and physicians worldwide have been observing individuals who developed multisystem symptoms and new-onset intolerances to xenobiotics, including formerly tolerated chemicals, foods, and drugs [32,33,44,45,46].

Based upon reports by researchers, physicians, and patients, Miller and Prihoda developed the Quick Environmental Exposure and Sensitivity Inventory (QEESI), a 50-item, internationally validated questionnaire designed to assess the symptoms, intolerances, and life impacts of chemical, food, and drug exposures. To date, researchers and clinicians in sixteen countries have used the QEESI, which offers high sensitivity and specificity for differentiating individuals with CI from the general population. Supplementary Figure S1 offers a comprehensive table and references for 96 international studies using the QEESI. Published in 1999, the QEESI was based on symptoms and intolerances reported by *groups* of individuals with well-characterized exposures to organophosphate pesticides, VOCs associated with new construction or remodeling, the Gulf War, and breast implants [47,48,49]. Again, at that time, there was no known biomechanism to explain these observations. Although mast cells were discovered more than one hundred years ago, their role in protecting our tissues from toxicants has only recently been described [50,51].

### 1.4. Toxicant-Induced Loss of Tolerance (TILT)

**Figure 1:**
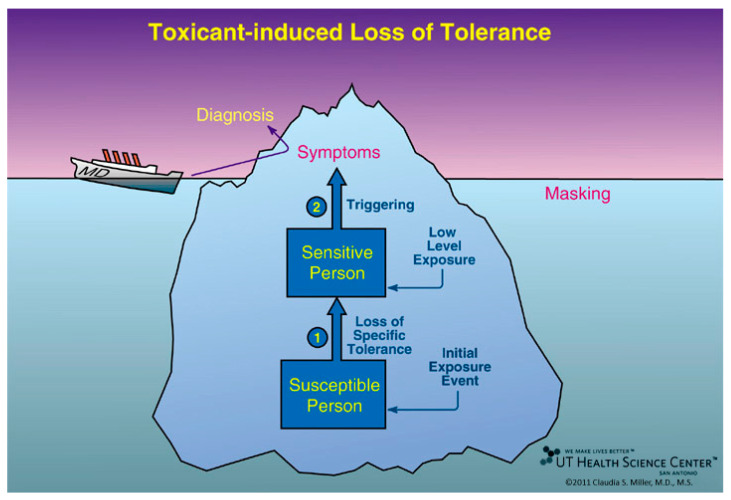
Toxicant-Induced Loss of Tolerance (TILT). *Initiation* is the first of two stages in the disease process, Toxicant-Induced Loss of Tolerance, or TILT. As shown here, *Initiation* (Stage 1 of TILT) involves a single major exposure or repeated exposures to toxicants such as pesticides, solvents, or toxic mold. In Stage 2 of TILT, called *Triggering*, tiny quantities of previously tolerated substances that never bothered the person before and do not bother most people trigger symptoms. Triggers often include diesel exhaust, cleaning products, fragrances, foods/food additives, drugs and their excipients, and food/drug combinations such as red wine, beer, coffee, or chocolate. A physician sees only the tip of the iceberg—the patient’s symptoms—and formulates a diagnosis based on them, e.g., asthma, ADHD, autism, or an autoimmune disorder. Background exposures “mask” or hide the relationship between symptoms and triggers. The initial exposure event that led to a loss of tolerance may go unrecognized. Adults may not recall *initiating* exposures that occurred during their childhood, for example, riding their bikes behind a truck spraying DDT, living where pesticides were applied, or residing in homes where coal, oil, natural gas/propane, or wood was used for heating or cooking [32,33].

**Figure 2:**
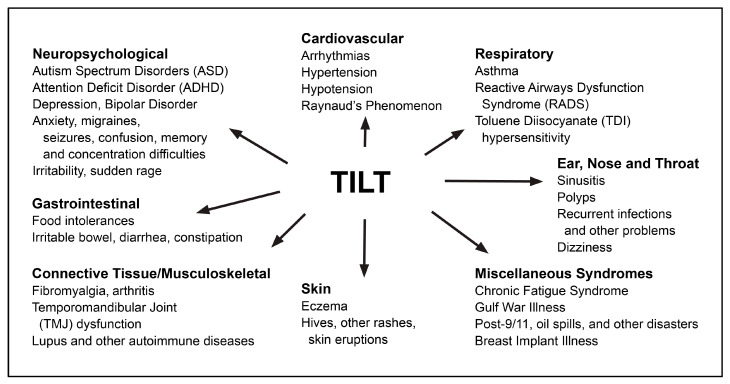
Conditions that may have their origins in TILT.

**Figure 3:**
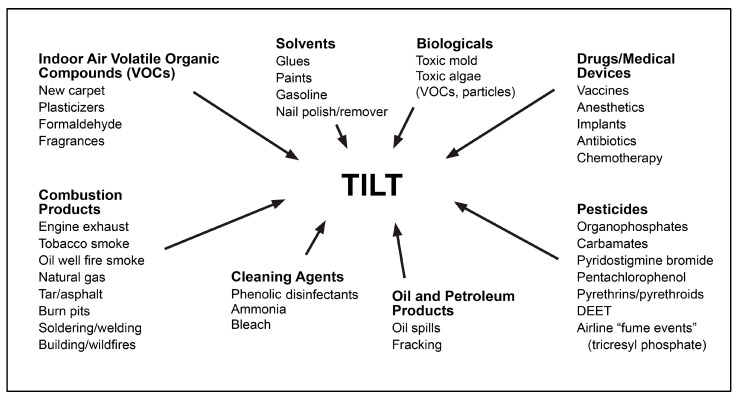
Potential initiators and triggers for TILT.

Toxicant-Induced Loss of Tolerance (TILT) (Figure 1) is a two-stage disease process involving *Initiation* by an exposure event (Stage 1), followed by *Triggering* (Stage 2) of symptoms by exposures to previously tolerated and often structurally unrelated chemical inhalants, ingestants, and medications [32,33,44]. A wide range of symptoms and medical conditions involving any and every organ system have been linked to TILT and mast cells (Figure 2) [32,33]. Large numbers of patients attribute the initiation of their illness to well-defined events such as exposures to pesticides, new construction or remodeling, indoor air contaminants, or a flood- or water-damaged building resulting in mold and bacterial growth (Figure 3) [49,52,53,54].

We have previously demonstrated that women with high CI scores on the QEESI have 3 times the risk of reporting a child with autism and 2.3 times the risk of having a child with ADHD compared to control mothers [31]. The present study further investigates the association between CI in parents and the risk of autism and ADHD in their children using a larger sample of U.S. adults.

## 2. Materials and Methods

This observational study involved a population-based survey of U.S. adults aged 18 years and older. SurveyMonkey recruitment procedures are available here: https://www.surveymonkey.com (accessed 17 February 2024). 10,981 respondents were randomly selected from nearly 3 million online users of the SurveyMonkey platform. The survey had an abandonment rate of 10.1% and took an average of 5 min to complete.

The modeled error estimate for this survey was ±1.4%. Respondents were selected from online panels based on the population sizes of all 50 states plus the District of Columbia, as well as by sex, age, race, and educational level within each census region to match the U.S. Census Bureau’s 2015 American Community Survey (ACS) targets. Of the 10,981 respondents, 4235 (40.07%) reported no biological children and were excluded from the study.

Respondents are classified into high or low CI groups, and autism/ADHD prevalence is calculated. It is important to note that the correlations identified in this study are not considered causal.

### 2.1. Survey

Respondents answered an 80-item survey we called the *Personal Exposure Inventory* (PEI), which included items concerning individuals’ demographics, medical diagnoses, and CI. Age and income were captured as part of SurveyMonkey’s panel. Age was reported as a four-level categorical variable, with age increasing roughly every 15 years. Income was reported as a ten-level categorical variable, with income increasing by roughly USD 25,000 per level (Table 1).

CI was assessed using the QEESI Chemical Exposures and Symptoms scales, which incorporate 0 to 10 severity ratings [47,48].

**Figure 4:**
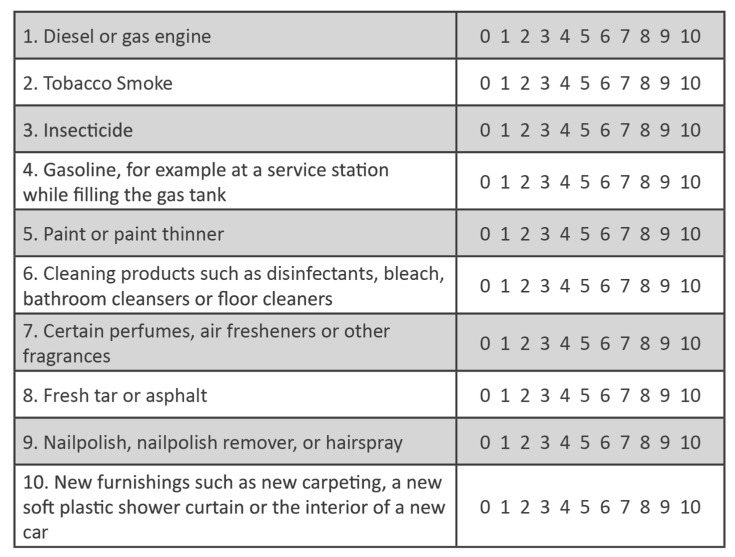
QEESI Chemical Exposures Scale. The sum of the symptom severity ratings for all 10 of these structurally unrelated chemical inhalants is the Total Chemical Intolerance score (0–100).

The QEESI’s Chemical Exposures Scale (Figure 4) asks participants to “indicate whether or not these odors or exposures would make you feel sick, for example, you would get a headache, have difficulty thinking, feel weak, have trouble breathing, get an upset stomach, feel dizzy, or something like that. For any exposure that makes you feel sick, on a 0–10 scale, rate the severity of your symptoms with that exposure” (0 = not at all a problem; 5 = moderate symptoms; 10 = disabling symptoms).

The severities of all 10 chemical inhalant items are added together to arrive at a Total Chemical Intolerance Score (0–100). The higher the Total Chemical Intolerance Score, the greater the likelihood that a person suffers from CI [47,48]. Note that the 10 items on the QEESI Chemical Exposure Scale were selected to be structurally/chemically diverse.

### 2.2. QEESI Scoring

The QEESI has four scales: Chemical Exposures, Other Exposures, Symptoms, and Life Impact. Each scale contains 10 items that are rated from 0 to 10 on a Likert scale: 0 = “not at all a problem” to 10 = “severe/disabling symptoms”. Total scores for each scale range from 0 to 100. Only the chemical and symptom scales were used to classify participants into CI severity groups [47,48]. The cut-off criteria for “High CI” are scores greater than or equal to 40 on both the chemical exposures and symptoms scales. “High CI” scores are considered to be “very suggestive” of CI. Scores from 20 to 39 on one or both scales are “suggestive” of CI. Scores less than 20 on both scales are “not suggestive” of CI.

To test our main hypothesis of a link between CI and autism/ADHD, respondents were asked: (1) “How many of your biological children have been diagnosed with autism, Asperger’s disorder, pervasive development disorder, or autism spectrum disorder by a doctor or health professional?” and (2) “How many of your biological children have been diagnosed with Attention Deficit Hyperactivity Disorder (ADHD) or Attention Deficit Disorder (ADD) by a doctor or health professional?”

### 2.3. Data Quality Control Checks

**Figure 5:**
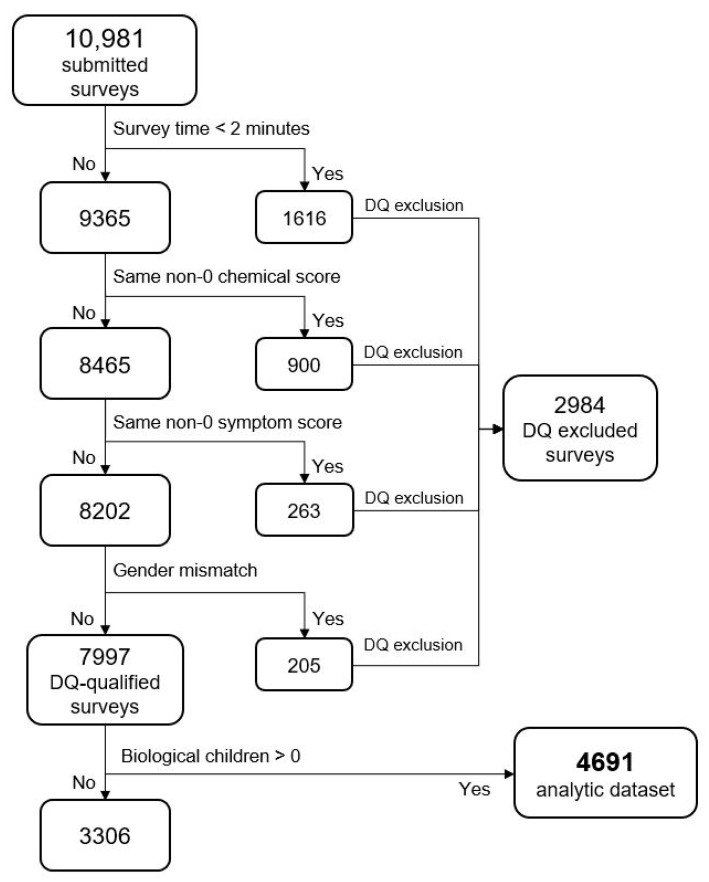
Data exclusion flow. Note: Same non-zero score means responses that had all the same number responses (e.g., all 1′s or all 2′s, etc.).

The 10,981 survey records were assessed for data quality (DQ) encompassing completeness, validity, or accuracy concerns; four measures were used to exclude surveys indicating one or more DQ concerns. Records with these concerns were excluded from the analytic data set. Figure 5 depicts the flow of data exclusions leading to the final analytic dataset. Some of the DQ measures might technically be accurate (e.g., “male and breast implants”), but with an abundance of caution, they were excluded. The same could be said for the “Too Fast” measure: with a survey containing a minimum of 29 questions, it is unlikely that a respondent could read and respond accurately to all questions in under two minutes. By omitting any records that violated one or more DQ measures, 2984 records were excluded (27.2%). We have taken this approach to help ameliorate some well-known DQ concerns associated with web-based surveys, including response probabilities and biases [55,56]. After applying both the data quality and the “no biological child” exclusions, our final analytic sample was N = 4691.

### 2.4. Statistical Modeling

A binary logistic regression was conducted to determine the extent to which parental CI was predictive of autism or ADHD in offspring in separate models. The binary dependent variable, “Reported autism”, was defined as any biological child of the respondent reported as having autism, Asperger’s disorder, pervasive development disorder, or autism spectrum disorder as diagnosed by a health professional. “Reported ADHD” was defined as any biological child of the respondent reported as having Attention Deficit Hyperactivity Disorder (ADHD) or Attention Deficit Disorder (ADD) diagnosed by a health professional.

The primary independent variable of interest, “High CI”, compared individuals with *very suggestive* QEESI CI scores (chemical exposures and symptom scores ≥ 40) to respondents with low CI scores (chemical exposures and symptom scores both ≤ 20). The middle or “suggestive” category was excluded from the analysis, creating a strong dichotomy between individuals clearly suffering from CI and those not exhibiting CI. The logistic regression model included age, sex, household income, and the number of children as independent variables. Although the dependent variable is at the family level, the number of children was included as a covariate (the more children, the more likely it is that at least one has autism/ADHD). After the middle “suggestive” category was excluded, the regression model N was 2038. A *p*-value of 0.05 and 95% confidence intervals were used to determine statistical significance. Analyses were conducted using SAS (version 9.4) and JMP (version 15) statistical software [57,58].

## 3. Results

**Figure 6:**
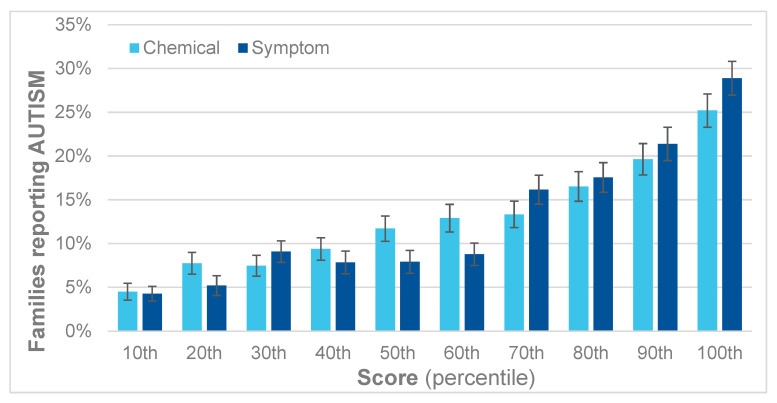
Percent of children with autism by decile for the QEESI total symptoms and chemical intolerance scores.

Figure 6 shows that the reported percentages of children with autism gradually increase with each decile (10 percentage points) for both the QEESI total chemical intolerance and symptoms scores. Comparing the highest to the lowest decile of the total chemical intolerance scores yields a relative risk (RR) of 5.7, and for the total symptoms scores, a RR of 7.1.

**Figure 7:**
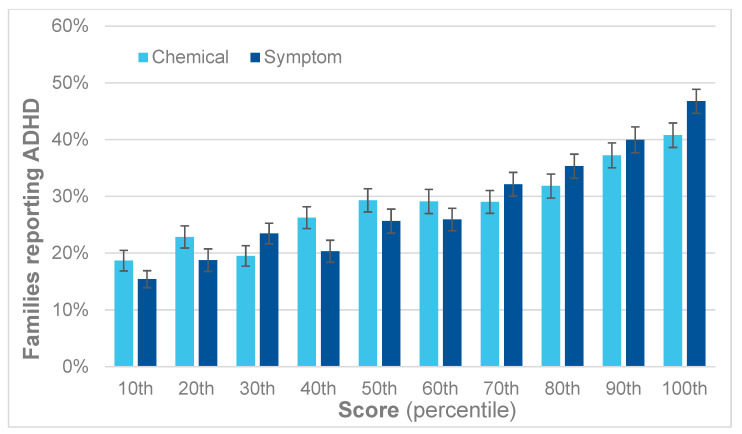
Percent of children with ADHD by decile for the QEESI total symptoms and chemical intolerance scores.

A similar trend is apparent for ADHD, as shown in Figure 7. For each decile, there is a corresponding increase in reported ADHD. Comparing the first and last deciles yields an RR for ADHD of 2.1 for the QEESI total chemical intolerance scores and 2.8 for the total symptoms scores.

Table 2 presents the risk ratios for each decile of the QEESI chemical intolerance score. Comparing the 100th to the 10th percentile, the RR is 5.7; comparing the 90th to the 10th, the RR is 4.1; and so on. Each RR is statistically significant but begins to decrease when the 30th percentile is compared to the 10th. Below that, the statistical significance becomes marginal.

As described above, in the past, we have used scores on both the chemical exposures and symptoms scales to determine Low, Mid, or High CI classifications.

**Figure 8:**
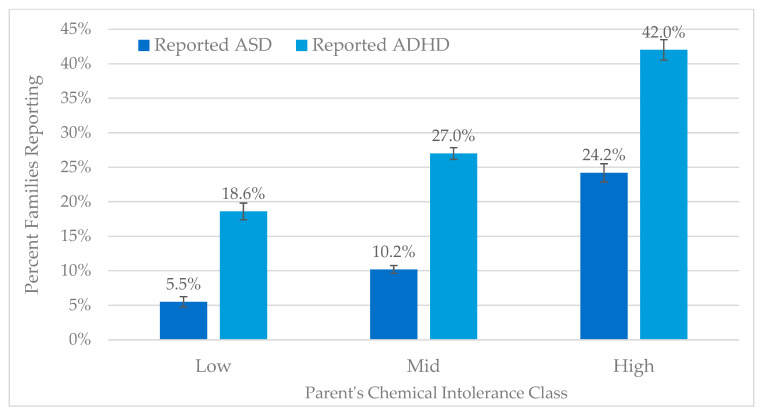
Reported autism and ADHD by QEESI Chemical Intolerance categories.

Figure 8 demonstrates that, relative to Low CI, individuals with High CI are more likely to report having a child with either autism (RR 4.2) or ADHD (2.3).

Table 3 shows the N for the percentages in Figure 8. 5.5% of parents in the low CI category report having a child with autism. 24.2% of parents in the high CI category report having a child with autism. A similar trend is observed for ADHD.

Since our original publication in 2015 [31], for which the QEESI was also used and Odds Ratios were calculated comparing High versus Low categories, there appears to have been an increase in the OR for both autism and ADHD: the Odds Ratio (OR) for autism has increased from 3.01 to 5.29, and the OR for ADHD, which was 2.3 in 2015, is now 3.18. The current paper presents RR not OR. Whether using RR or OR, the present paper reveals increased risks for both autism and ADHD in recent years.

## 4. Discussion

In an earlier study, nearly half of the respondents we studied reported developing CI after one or more toxic exposures [59]. The most frequently cited initiating exposures were mold (15.6%), pesticides (11.5%), medical/surgical procedures (11.3%), remodeling/new construction (10.7%), fires/combustion products (6.4%), and implants (1.6%). In addition, protracted antibiotic use for difficult-to-treat-infections involving the prostate, skin, tonsils, gastrointestinal tract, and sinuses, was strongly associated with TILT/CI (OR > 2) [59]. Survey participants identified two broad classes of TILT initiators: (1) *fossil fuel-derived toxicants* from coal, natural gas, oil, their combustion products, and/or synthetic chemical derivatives such as pesticides, implants, drugs/antibiotics, VOCs, endocrine disruptors (EDs), persistent organic pollutants (POPs), or (2) *biogenic toxicants* including particles and VOCs from toxic mold or toxic algae [53,59].

The U.S. government sets “safe exposure levels”, most often based on animal testing [60]. These levels are referred to as “No Observed Adverse Effect Levels” or NOAELs. Federal agencies, including the EPA, OSHA, NIOSH, NIEHS, FDA, and others, follow these guidelines. Major limitations of NOAELs include the fact that they do not apply to chemical mixtures, carcinogens, or mutagens. In addition, dose–response testing results in recommended levels that are far too high to protect people whose mast cells have been sensitized, in particular, individuals with CI.

### 4.1. Indoor vs. Outdoor Air

Many people believe that the air inside their homes is relatively “clean”. However, particles and gases tend to concentrate in enclosed spaces, making indoor air more hazardous than outdoor air. Indoor air VOCs released by solvents, cleaning chemicals, and fragrances are common TILT initiators and triggers. We spend most of our day inside homes, schools, workplaces, cars, buses, trains, etc., where chemicals from many sources are released and can accumulate to high levels. Other common indoor VOC sources include outgassing from plastics such as new shower curtains, upholstery, furnishings, carpeting, and construction materials [34,59].

### 4.2. Toxicant-Induced Epigenetic Changes Could Explain CI and the Heritability of Autism and ADHD

Epigenetics and toxicogenetics (or toxicogenomics) are rapidly growing, overlapping fields [61]. Both fields have established the role of an individual’s personal environment in altering genes involved in a wide variety of medical conditions [62,63]. Substantial epidemiological literature links toxic exposures and genetics to autism [64,65]. A plethora of xenobiotics target millions of different genes. Gene/Environment (GxE) interactions are now considered the best explanation for *idiopathic autism*, which represents the vast majority of cases (in only 4–20% of cases has a specific cause been identified) [6,9,66]. Various reports indicate that immune dysfunction and increased inflammatory cytokines in children and mothers are associated with autism [67,68,69]. During pregnancy and early childhood, all organ systems are potential targets for toxic exposures (respiratory, skin, liver, kidney, cardiovascular, reproductive, hematologic, and neurological).

### 4.3. TILT and Mast Cells as a Plausible Biomechanism for Autism and ADHD

Only in the past two decades have scientists begun to understand mast cells and their function as the “first responders” in our immune systems. In earlier papers, Miller et al. (2021) proposed that toxic exposures can alter mast cells which subsequently respond erratically to formerly well-tolerated xenobiotics, including common chemicals, foods, and drugs. Protecting parents and their offspring from toxicants and identifying xenobiotics (chemicals, foods, and drugs) that can trigger symptoms may prove essential for reducing the incidence of autism and ADHD [50,59].

Mast cells first appeared more than 500 million years ago in early vertebrate fish, evolving into neuroimmunoendocrine cells and eventually into master regulators effecting neuroinflammation [70,71]. They are specialized white blood cells that originate in the bone marrow and migrate to the interfaces between all of our tissues, including the blood–brain barrier, and the external environment, e.g., the airways, digestive tract, skin, urogenital tract, and lymphatic and blood vessels. In the nose, sensitized mast cells can be triggered by low-level exposures such as diesel exhaust, tobacco smoke, pesticides, or fragrances. When triggered, they release complex cascades containing nearly 400 inflammatory mediators, which affect physiological, immunological, and inflammatory processes [70,71,72,73].

An important factor affecting immunogenicity is molecular weight. If the molecular weight of a foreign chemical (xenobiotic) is less than 10,000 Daltons, the mast cell will initiate cell-mediated immunity (CMI), also known as delayed-type hypersensitivity (DTHS) [74]. The majority of xenobiotics weigh far less than 10,000 Daltons, for example, pesticides, plasticizers, dioxins, fragrances, food proteins and carbohydrates, food additives, MSG, caffeine, etc. If toxicants epigenetically “turn on” or “turn off” genes that are essential for normal mast cell development and function, this could readily explain our findings of increased autism and ADHD in the offspring of parents who have developed chemical intolerance. The epigenetic consequences of acute, repeated, or chronic exposures could be anticipated to be inappropriate or erratic responses by mast cells to previously tolerated xenobiotics, that is, intolerances for chemicals, foods, and drugs—precisely the mechanism of disease Miller first described in the late 1990s as “Toxicant-Induced Loss of Tolerance” or TILT [33,49]. Prolonged inflammation triggered by xenobiotics may explain the connection between CI and inflammation in the brain, which characterizes autism [75,76].

### 4.4. Autism Intervention and Support

There are both pharmacologic and non-pharmacologic approaches for treating autism. Currently, Applied Behavioral Analysis (ABA) is considered the most effective and widely used non-pharmacologic intervention. However, it is not effective for everyone, and treatments should be individualized [77,78,79]. As Dr. Stephen Shore said, “If you’ve met one person with autism, you’ve met one person with autism”. Individuals with autism present unique strengths and difficulties and experience the disability in different ways [80].

Drug therapies are often used to address adverse behaviors and comorbidities such as sleep difficulties and anxiety that are not controlled by behavioral therapies. Aripiprazole and risperidone are currently approved for the treatment of autism and can alleviate self-aggressive, angry, and/or irritable behaviors [80]. Medications such as cromolyn, which helps stabilize mast cells, and H_1_ and H_2_ antihistamines, which block the effects of mast cell inflammatory mediators on tissues, appear to be useful for treating at least some individuals with autism and ADHD [75,76,81,82,83].

### 4.5. Prevention

Improving policies designed to protect workers and communities from chemical spills, releases, or fires has great potential for risk reduction. Personal choices are important too. For example, avoiding the use of artificial sweeteners during pregnancy has recently been shown to increase the risk of autism in boys threefold [84]. Our current and prior autism and ADHD studies suggest that using the QEESI to gauge CI in prospective parents and teaching them how to reduce their exposures to toxicants such as pesticides and fragrances may help reduce their risk of having a child with a neurodevelopmental disorder [31]. We counsel many patients and their families who are dealing with CI, asthma, and other conditions to substitute products or change practices. For example, instead of using pesticides indoors, one can use baits or traps to eliminate pests. Individuals who are pregnant or hoping to have a child should take the QEESI to gauge their risk of having a child with autism or ADHD. They can be counseled and assisted in reducing personal exposures that may adversely affect neurodevelopment.

Because people in industrialized countries typically spend 90% or more of their day indoors, special attention must be given to home, work, and school environments. We encourage patients to keep the air inside their homes as free as possible from chemicals, smoke, fragrances, and allergy triggers. Our website, TILTresearch.org (accessed 17 February 2024) offers “7 Steps to Creating a Clean Air Oasis” (Figure S1). In addition, we have developed a “TILT Tutorial on Chemical Intolerance, Autism, and ADHD”, which is also available on our website. The tutorial describes the need for hospital-based Environmental Medical Units (EMUs) to help patients with severe CI or autism; what employers, administrators, property owners, and schools can do; and how doctors and other health professionals can use the QEESI to identify/prevent CI and autism/ADHD.

By using the QEESI and applying our knowledge of the role of toxicants in autism and ADHD, current and future autism treatment centers can help teach prospective parents how they might prevent these conditions in their children. Autism treatment centers might be reframed as “autism *prevention* and treatment centers”, incorporating the best preventive practices and designed and operated as model facilities that minimize potentially toxic exposures. It is now clear that personal exposure to toxicants can adversely affect neurodevelopment. Therefore, we must do everything in our power to prevent these exposures from happening in the first place. We can predict and prevent autism and ADHD by using the QEESI and targeting CI.

### 4.6. Study Limitations

The limitations of this study fall into three broad categories: (1) Survey Methodology, (2) Missing Information, and (3) Autism/ADHD Definition.

#### 4.6.1. Survey Methodology

As indicated in the Section 2, the overall study design is observational, and no causality can be established without further research. The survey was conducted via a paid, computerized survey platform (SurveyMonkey). As such, all respondent answers were self-reported and therefore prone to several biases, including social acceptability, honesty, differing interpretations of questions, and recall bias. Payments to participants were small (less than USD 10) and did not constitute “undue influence”. To address both payment and self-report concerns, extensive data quality procedures were employed to remove surveys completed too quickly or illogically.

Although the survey was balanced to reflect state population sizes, participants’ sex, age, race, and education, selection bias in computer-based surveys can be marked. Our computerized surveys suggest under-sampling of Blacks/African Americans and Hispanics/Latinos, both by nearly 50%. Despite concerns about under-sampling of elderly subjects due to computer literacy/access, the survey actually over-sampled respondents 45 years of age and older and under-sampled younger respondents (18–44 years old). Lack of access to the Internet, a computer, or a smartphone, as well as language limitations, may have also reduced the generalizability of our findings for low-income and minority populations.

#### 4.6.2. Missing Information

Missing information limited the explanatory power of the statistical models used in this study. First, the absence of race/ethnicity data for our participants prevents any comparison of CI, autism, or ADHD prevalence across different minorities. Second, not obtaining data concerning a child’s sex, a known factor in the prevalence of these conditions, creates a larger residual error in statistical modeling. The impetus for this study was to gather population-level estimates of CI. Consequently, modeling factors related to autism/ADHD were not considered in the survey design. Subsequent analysis of CI and autism survey data indicates the need for future, closer examination with appropriate covariates.

#### 4.6.3. Autism/ADHD Definition

Autism was determined by parental self-reports of the number of biological children diagnosed with autism or ADHD. As such, possible self-report biases should be recognized. However, research has indicated that parental reports of autism are quite accurate [85,86]. The age or sex of these children was not recorded. Thus, comparisons to other autism prevalence estimates, e.g., from the CDC, are not appropriate.

## 5. Conclusions

This study, together with our previously published study [31], provides strong evidence that CI is a risk factor for autism and ADHD. TILT appears to be initiated by toxic exposures resulting in mast cell alteration, potentially epigenetic, and subsequent mast cell activation. Thereafter, structurally diverse xenobiotics, including chemicals, foods, and drugs that never bothered the person previously and do not bother most people, trigger multisystem symptoms that wax and wane over time. Persistent activation and triggering of mast cells may underlie the brain inflammation in autism. The potential role of environmental toxicants in influencing epigenetics and mast cell function is a complex and emerging area of research. The implications of this study, if confirmed, could be significant for preventive measures and early intervention strategies in families with parental chemical intolerance. We recommend that all prospective parents be assessed for CI at an early age. Primary care physicians, as well as psychiatrists, psychologists, and social workers who care for individuals at increased risk, need to understand and communicate the far-reaching consequences of CI. They should screen patients and prospective parents using the QEESI, refer as appropriate, and emphasize the importance of reducing TILT initiators and triggers such as pesticides, fragrances, and tobacco smoke, particularly during pregnancy and childhood.

Acknowledging the need for further evidence, we hope this study contributes to an improved understanding of the potential role of environmental factors in the global rise of autism and ADHD. To assist with this process, healthcare professionals and their patients can access the free TILT Tutorial on Autism and ADHD at our website, https://TILTresearch.org (accessed 17 February 2024) under “Resources and Links” where they will find practical steps for screening patients and reducing potentially hazardous exposures.

Recommendations for outreach, education, and future research:

1. Increase awareness of autism/ADHD prevention by assessing CI using the QEESI.
2. Conduct population-based surveys to determine the prevalence of CI in other populations, countries, and regions.
3. Fund research to improve prevention, and environmental and medical interventions related to CI, TILT, mast cells, and autism/ADHD.

We invite patients, practitioners, and other researchers to take advantage of the free tools we have created, which include the QEESI, Brief Exposure History, “7 Steps to Creating a Clean Indoor Air Oasis”, and the QEESI Symptom Star, which can be used to graphically illustrate individual or group symptoms pre- and post- an exposure event, as well as pre- and post-environmental and/or medical interventions. Our “TILT Tutorial on Chemical Intolerance, Autism, and ADHD”, available at https://TILTresearch.org (accessed 17 February 2024), describes these resources. We look forward to research by other investigators whose work may confirm, extend, or challenge our findings.

## Figures and Tables

**Table 1:** Demographic characteristics of the sample.

		Percent
Age	1: 18 to 29 years	10.2
	2: 30 to 44	23.1
	3: 45 to 60	39.1
	4: 61+	27.7
Sex	Male	40.5
	Female	59.5
Household Income	1: USD 0–9999	3.9
	2: USD 10,000–24,999	9.5
	3: USD 25,000–49,999	18.8
	4: USD 50,000–74,999	20.1
	5: USD 75,000–99,999	14.0
	6: USD 100,000–124,999	9.8
	7: USD 125,000–149,999	5.9
	8: USD 150,000–174,999	3.1
	9: USD 175,000–199,999	2.0
	10: USD 200,000+	4.4
	Prefer not to answer (Missing)	8.5
Number of Children	1	28.3
	2	39.2
	3	18.4
	4	6.3
	5	2.1
	6	1.7
	Missing	4.1
High Chemical Intolerance Classification		22.6
Families reporting autism		13.0
Families reporting ADHD		28.6

**Table 2:** Risk ratios for deciles with the 10th percentile as the referent group.

Percentile	Chemical Intolerance Score (0–100)	Any Reported Autism		Any Reported ADHD	
		Risk Ratio (Compared to the 10th)	95% Confidence Interval	Risk Ratio (Compared to the 10th)	95% Confidence Interval
100th	>63	5.7 ***	[3.57, 9.08]	2.1 ***	[1.70, 2.63]
90th	53 to 63	4.1 ***	[2.55, 6.69]	1.9 ***	[1.53, 2.40]
80th	45 to 52	3.7 ***	[2.30, 6.09]	1.7 ***	[1.33, 2.13]
70th	38 to 44	3.0 ***	[1.83, 4.96]	1.5 ***	[1.20, 1.93]
60th	31 to 37	2.8 ***	[1.71, 4.72]	1.5 ***	[1.18, 1.91]
50th	25 to 30	2.8 ***	[1.72, 4.69]	1.6 ***	[1.24, 1.98]
40th	18 to 24	2.1 **	[1.25, 3.54]	1.4 **	[1.08, 1.75]
30th	12 to 17	1.7	[0.99, 2.95]	1.0	[0.79, 1.34]
20th	6 to 11	1.7	[0.96, 2.89]	1.2	[0.92, 1.53]
10th	<6	1.0	[0.54, 1.81]	1.0	[0.76, 1.31]

** *p* ≤ 0.01; *** *p* ≤ 0.001. Note: These risk ratios compare chemical intolerance scores for individuals in each percentile against scores for the bottom 10th percentile. For example, individuals whose QEESI CI scores are greater than 61 have 5.7 times the risk of having a child with autism and 2.2 times the risk of having one with ADHD, compared to individuals whose QEESI CI scores are below the 10th percentile, that is, less than 7. Significant risk is indicated above the 30th percentile.

**Table 3:** N’s for autism and ADHD by CI class.

Chemical Intolerance Class	Autism				ADHD			
	Families Not Reporting Autism	Families Reporting Autism	All	%	Families Not Reporting ADHD	Families Reporting ADHD	All	%
	N	N	N		N	N	N	
Low	948	55	1003	5.5%	816	187	1003	18.6%
Mid	2378	271	2649	10.2%	1934	715	2649	27.0%
High	788	251	1039	24.2%	608	431	1039	41.5%

## Data Availability

The datasets for the current study are available from the corresponding author upon reasonable request.

## Supplementary Materials

The following supporting information can be downloaded at: https://www.mdpi.com/article/10.3390/jox14010022/s1, Figure S1: 7 Steps to Creating a Clean Air Oasis. Table S1: Peer-reviewed Journal Articles Using the QEESI by Country.
