# Supplementary material for: Assessing Chemical Intolerance in Parents Predicts the Risk of Autism and ADHD in Their Children
Source: J Xenobiot. 2024 Mar 5;14(1):350–67. doi: 10.3390/jox14010022 (PMC10970846; doi:10.3390/jox14010022)
Supplement: Supplementary file 1 [file jox-14-00022-s001.zip › jox-2825343-supplementary.pdf]

Journal of Xenobiotics

MDPI

Supplementary Materials: Assessing Chemical Intolerance in Parents Predicts the Risk of Autism and ADHD in Their Children

Raymond F. Palmer, David Kattari, Rodolfo Rincon and Claudia S. Miller

7 Steps to Creating a Clean Air Oasis

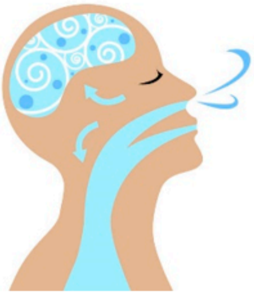

We spend 90% of our day indoors where the air often is more polluted than the air outside.

Research suggests that a "clean room" may help people who suffer from breathing difficulties, allergies, headaches, brain fog/confusion, fatigue, and other health problems.

You can create a clean air oasis in your home, or in one room, where the air is as free as possible of chemicals, smoke, fragrances, and allergy triggers.

1. **Pick a room**
   Choose the room where you spend most of your time, usually your bedroom. Bring in clean outside air whenever possible, but avoid opening windows when pollen or pollution levels are high.

2. **Eliminate indoor air pollutants:** Remove all products that have strong odors including cleaning and laundry products, pesticides, perfume/cologne, scented lotions, deodorants, cosmetics, candles, air fresheners (including plug-ins and diffusers). Avoid any aerosol sprays (such as hair spray) because their tiny droplets are easily inhaled.

3. **Ask your primary care doctor about allergy testing:** Dander (dead skin cells) from pets, dust mites, mold, and seasonal pollens can trigger asthma, allergies, and other problems.

4. **Clean safely :** Use only fragrance-free products for cleaning and doing laundry. Cleaning and vacuuming are best done when sensitive individuals are not in the immediate area. Ventilate during and after cleaning. See list of cleaning supplies and instructions on the back of this page.

5. **Avoid burning anything indoors:** Smoke and combustion gases irritate the lungs. Do not permit smoking, vaping, or the burning of candles or incense. Do not use fireplaces, open-flame gas heaters, or unvented water heaters. Prevent carbon monoxide poisoning—never heat your home using a gas stove, gas oven, or Hibachi. If you move or purchase new appliances, electric stoves and other electric appliances are the better health option.

6. **Go the extra mile:** An air purifier with HEPA and charcoal filters can remove some pollutants. Keep the purifier running while the room is occupied, including overnight. Bring in clean outside air whenever possible.

7. **Learn more:** Many of our choices affect the quality of air we breathe. Learn how to remove fragrances from fabrics, stop pests without using pesticides, control humidity and mold, and find safer products for home repair/remodeling. *Visit TILTresearch.org for more information.*

UT Health
San Antonio
Family & Community Medicine

Figure S1. 7 Steps to Creating a Clean Air Oasis at Home.

Table S1: **Peer-reviewed Journal Articles Using the QEESI by Country**

COUNTRY	AUTHOR & DATE	N		COUNTRY	AUTHOR & DATE	N
**Austria**	Weiss, 2017	72		**Japan**	Hojo, 2009	412
**Colombia**	Storino, 2021	1		**Japan**	Ishibashi, 2007	214
**Denmark**	Dantoft, 2014	298		**Japan**	Lu, 2020	667
**Denmark**	Hauge, 2015	69		**Japan**	Manabe, 2008	368
**Denmark**	Skovbjerg, 2012	1493		**Japan**	Mizuki, 2004	32
**Denmark**	Tran, 2014	3		**Japan**	Mizuki, 2015	40
**Denmark**	Tran, 2017	39		**Japan**	Mizukoshi, 2015	8
**Finland**	Heinonen-Guzejev, 2012	327		**Japan**	Nakaoka, 2018	43
**Finland**	Selinheimo, 2019	52		**Japan**	Ohsawa, 2020	2
**Finland**	Vuokko, 2019	12		**Japan**	Suzuki, 2020	141
**Finland**	Suojalehto, 2021	88		**Japan**	Watai, 2018	528
**Finland**	Nynäs, 2021	667		**Japan**	Yoshino, 2004	69
**Finland**	Karvala, 2018	4971		**Saudi Arabia**	Khalil, 2020	134
**Finland**	Suojalehto, 2021	88		**South Korea**	Heo, 2017	1030
**France**	Kamoun, 2011	20		**South Korea**	Jeon, 2012	300
**Germany**	Bauer, 2007	202		**South Korea**	Jeong, 2014	379
**Germany**	Schnakenberg, 2007	521		**South Korea**	Yun, 2013	1
**Indonesia**	Hildebrandt, 2019	471		**South Korea**	Woo Kwon, 2019	261
**Indonesia**	Kubota,2020	707		**Spain**	Aguilar-Aguilar, 2018	52
**Indonesia**	Sani, 2023	896		**Spain**	Alobid, 2014	118
**Italy**	Caccamo, 2013	443		**Spain**	Fernandez-Solà, 2005	75
**Italy**	De Luca, 2010	444		**Spain**	García-Sierra, 2014	125
**Italy**	De Luca, 2014	300		**Spain**	Lago Blanco, 2016	73
**Italy**	De Luca, 2015	563		**Spain**	Mena, 2013	231
**Italy**	Gugliandolo, 2016	34		**Spain**	Nogué, 2007	52
**Italy**	Micarelli, 2016a	38		**Spain**	Paredes-Rizo, 2018	1
**Italy**	Micarelli, 2016b	38		**Spain**	Pérez-Crespo, 2018	514
**Italy**	Viziano, 2017	38		**Spain**	Loria KohenV, 2017	52
**Italy**	Cannata, 2021	38		**Spain**	Fares-Medina 2022	210
**Japan**	Azuma, 2021	4683		**Sweden**	Andersson, 2009	207
**Japan**	Azuma, 2013	122		**Sweden**	Nordin, 2010	283
**Japan**	Azuma, 2015a	7245		**Taiwan**	Huang, 2011a	658

**Japan**	Azuma, 2015b	12		**Taiwan**	Huang, 2011b	tbd
**Japan**	Azuma, 2016	16		**Taiwan**	Huang, 2014	658
**Japan**	Azuma, 2019	909		**Taiwan**	Huang, 2015a	30
**Japan**	Cui, 2013	324		**Taiwan**	Huang, 2015b	30
**Japan**	Cui, 2014	2464		**United States**	Gould Peek, 2015	563
**Japan**	Cui, 2015	565		**United States**	Heilbrun, 2015	694
**Japan**	Fujimori, 2012	1,084		**United States**	Katerndahl, 2012	400
**Japan**	Hasegawa, 2009	51		**United States**	Miller, 1999a	421
**Japan**	Hojo, 2002	1260		**United States**	Miller, 1999b	421
**Japan**	Hojo, 2003	760		**United States**	Palmer, 2020	10,000
**Japan**	Hojo, 2005	440		**United States**	Masri, 2021	N/A
**Japan**	Hojo, 2008	106		**United States**	Palmer, 2020	293
**Japan**	Hojo, 2019	555		**United States**	Miller, 2021	147
**Japan**	Hojo, 2009	412		**Uruguay**	De Ben, 2014	2
**Japan**	Hojo, 2018	214				
**Japan**	Hojo, 2018	214				
**Japan**	Hojo, 2019	555				
**Japan**	Tsumura, 2023	149			**96 Publications**	
**Japan**	Nakaono, 2022	1		***16 Countries***	***Total N > 56,043 subjects***	

*Quick Environmental Exposure and Sensitivity Inventory (QEESI) Publications by Country

Austria

Weiss, E. M., Singewald, E., Baldus, C., Hofer, E., Marksteiner, J., Nasrouei, S., . . . Holzer, P. (2017). Differences in psychological and somatic symptom cluster score profiles between subjects with Idiopathic environmental intolerance, major depression and schizophrenia. *Psychiatry Research, 249*, 187-194. doi:10.1016/j.psychres.2016.12.057

China

Huang, L.-L., Ikeda, K., Chiang, C.-M., Kagi, N., Hojo, S., & Yanagi, u. (2011). Field Survey on the Relation between IAQ and Occupants' Health in 40 Houses in Southern Taiwan. *Journal of Asian Architecture and Building Engineering, 10*. doi:10.3130/jaabe.10.249

Huang, L.-l., Ikeda, K., Hojo, S., Kagi, N., & Juan, H.-C. (2014). Study of the different Cutoff Point of the QEESI Questionnaire as a Screening Tool for Sick Building Syndrome Diagnosis in Taiwan. *Journal of Asian Architecture and Building Engineering, 13*(2), 507-513. doi:10.3130/jaabe.13.507

Colombia

Storino, V., Muñoz-Ortiz, J., Villabona-Martinez, V., Villamizar-Sanjuán, J. D., Rojas-Carabali, W., & dela-Torre, A. (2021). An Unusual Case of Multiple Food Allergies Comorbid with Multiple Chemical Sensitivity: A Case Report. *Journal of asthma and allergy, 14*, 317–323. https://doi.org/10.2147/JAA.S293248

Denmark

Dantoft, T. M., Elberling, J., Brix, S., Szecsi, P. B., Vesterhauge, S., & Skovbjerg, S. (2014). An elevated prohttps://pubmed.ncbi.nlm.nih.gov/24485486/inflammatory cytokine profile in multiple chemical sensitivity. *Psychoneuroendocrinology, 40*, 140-150. doi:10.1016/j.psyneuen.2013.11.012

Hauge, C. R., Rasmussen, A., Piet, J., Bonde, J. P., Jensen, C., Sumbundu, A., & Skovbjerg, S. (2015). Mindfulness-based cognitive therapy (MBCT) for multiple chemical sensitivity (MCS): Results from a randomized controlled trial with 1 year follow-up. *Journal of Psychosomatic Research, 79*(6), 628-634. doi:10.1016/j.jpsychores.2015.06.010

Skovbjerg, S., Berg, N. D., Elberling, J., & Christensen, K. B. (2012). Evaluation of the Quick Environmental Exposure and Sensitivity Inventory in a Danish Population. *Journal of Environmental and Public Health, 2012*, 304314. doi:10.1155/2012/304314

Tran, M. T. D., Skovbjerg, S., Arendt-Nielsen, L., Bech, P., Lunde, M., & Elberling, J. (2014). Two of three patients with multiple chemical sensitivity had less symptoms and secondary hyperalgesia after transcranially applied pulsed electromagnetic fields. *Scand J Pain, 5*(2), 104-109. doi:10.1016/j.sjpain.2013.11.008

Tran, M. T. D., Skovbjerg, S., Arendt-Nielsen, L., Christensen, K. B., & Elberling, J. (2017). A randomised, placebo-controlled trial of transcranial pulsed electromagnetic fields in patients with multiple chemical sensitivity. *Acta Neuropsychiatrica. Officieel Wetenschappelijk Orgaan van Het IGBP (Interdisciplinair Genootschap voor Biologische Psychiatrie), 29*(5), 267-277. doi:10.1017/neu.2016.51

Finland

Heinonen-Guzejev, M., Koskenvuo, M., Mussalo-Rauhamaa, H., Vuorinen, H., Heikkil‰, K., & Kaprio , J. (2012).
Noise sensitivity and multiple chemical sensitivity scales: Properties in a population based epidemiological study. *Noise and Health, 14*(60), 215-223. doi:10.4103/1463-1741.102956

Selinheimo, S., Vuokko, A., Hublin, C., Järnefelt, H., Karvala, K., Sainio, M., . . . Paunio, T. (2019). Health-related quality among life of employees with persistent nonspecific indoor-air-associated health complaints.
Journal of Psychosomatic Research, 122, 112-120. doi:10.1016/j.jpsychores.2019.03.181

Vuokko, A., Karvala, K., Suojalehto, H., Lindholm, H., Selinheimo, S., Heinonen-Guzejev, M., . . . Sainio, M.
(2019). Clinical Characteristics of Disability in Patients with Indoor Air-Related Environmental
Intolerance. *Safety and health at work, 10*(3), 362-369. doi:10.1016/j.shaw.2019.06.003

Suojalehto, H.; Ndika, J.; Lindström, I.; Airaksinen, L.; Karvala, K.; Kauppi, P.; Lauerma, A.; Toppila-Salmi, S.; Karisola, P.; Alenius, H. Transcriptomic Profiling of Adult-Onset Asthma Related to Damp and Moldy Buildings and Idiopathic Environmental Intolerance. Int. J. Mol. Sci. 2021, 22, 10679. https://doi.org/10.3390/ ijms221910679

Nynäs, P.; Vilpas, S.; Kankare, E.; Karjalainen, J.; Lehtimäki, L.; Numminen, J.; Tikkakoski, A.; Kleemola, L.; Huhtala, H.; Uitti, J. Multiple Chemical Sensitivity in Patients Exposed to Moisture Damage at Work and in General Working-Age Population—The SAMDAW Study. Int. J. Environ. Res. Public Health 2021, 18, 12296. https://doi.org/10.3390/ ijerph182312296

France

Kamoun, H., Romdhane, N. A., Rekik, W., Laadheri, N., Youssef, I., Ben Salah, F., & Gharbi, R. (2011). Multiple chemical sensitivity syndrome. Observation of a cohort of 20 workers. *Archives des Maladies Professionnelles et de l'Environnement, 72*(1), 73-79. https://doi.org/10.1016/j.admp.2010.08.006

Germany

Bauer, A., Schwarz, E., Martens, U. (2007). Patients with multiple chemical sensitivities: A case for environmental or psychosomatic medicine? Zeitschrift fur Allgemeinmedizin, 83(11):442-446.

Schnakenberg, E., Fabig, K. R., Stanulla, M., Strobl, N., Lustig, M., Fabig, N., & Schloot, W. (2007). A cross-sectional study of self-reported chemical-related sensitivity is associated

with gene variants of drug-metabolizing enzymes. *Environmental health : a global access science source*, *6*,
6. https://doi.org/10.1186/1476-069X-6-6

Indonesia

Hildebrandt, S., Kubota, T., Sani, H. A., & Surahman, U. (2019). *Indoor Air Quality and Health in Newly Constructed Apartments in Developing Countries: A Case Study of Surabaya, Indonesia*. *Atmosphere*. 10(4):182

Kubota, T., Sani, H. A., Hildebrandt, S., & Surahman, U. (2020). Indoor air quality and self-reported multiple chemical sensitivity in newly constructed apartments in Indonesia. *Architectural Science Review*.
https://doi.org/10.1080/00038628.2020.1779647

Italy

Caccamo, D., Cesareo, E., Mariani, S., Raskovic, D., Ientile, R., Currò, M., . . . De Luca, C. (2013). Xenobiotic sensor- and metabolism-related gene variants in environmental sensitivity-related illnesses: a survey on the Italian population. *Oxidative Medicine and Cellular Longevity*, *2013*, 831969.
doi:10.1155/2013/831969

De Luca, C., Scordo, M. G., Cesareo, E., Pastore, S., Mariani, S., Maiani, G., . . . Korkina, L. G. (2010). Biological definition of multiple chemical sensitivity from redox state and cytokine profiling and not from polymorphisms of xenobiotic-metabolizing enzymes. *Toxicology and Applied Pharmacology*, *248*(3), 285-292. doi:10.1016/j.taap.2010.04.017

De Luca, C., Thai, J. C., Raskovic, D., Cesareo, E., Caccamo, D., Trukhanov, A., & Korkina, L. (2014). Metabolic and genetic screening of electromagnetic hypersensitive subjects as a feasible tool for diagnostics and intervention. *Mediators of Inflammation*, *2014*, 924184. doi:10.1155/2014/924184

De Luca, C., Gugliandolo, A., Calabrò, C., Currò, M., Ientile, R., Raskovic, D., . . . Caccamo, D. (2015). Role of
Polymorphisms of Inducible Nitric Oxide Synthase and Endothelial Nitric Oxide Synthase in Idiopathic
Environmental Intolerances. *Mediators of Inflammation*, *2015*, 245308. doi:10.1155/2015/245308

Gugliandolo, A., Gangemi, C., Calabrò, C., Vecchio, M., Di Mauro, D., Renis, M., . . . Caccamo, D. (2016). Assessment of glutathione peroxidase-1 polymorphisms, oxidative stress and DNA damage in sensitivity-related illnesses. *Life Sciences*, *145*, 27-33. doi:10.1016/j.lfs.2015.12.028

Micarelli, A., Viziano, A., Bruno, E., Micarelli, E., & Alessandrini, M. (2016a). Vestibular impairment in Multiple Chemical Sensitivity: Component analysis findings. *Journal of Vestibular Research*, *26*(5-6), 459-468. doi:10.3233/ves-160594

Micarelli, A., Viziano, A., Genovesi, G., Bruno, E., Ottaviani, F., & Alessandrini, M. (2016b). Lack of contralateral suppression in transient-evoked otoacoustic emissions in multiple chemical sensitivity: a clinical correlation study. *Noise Health, 18*(82), 143-149. doi:10.4103/1463-1741.181997

Viziano, A., Micarelli, A., & Alessandrini, M. (2017). Noise sensitivity and hyperacusis in patients affected by multiple chemical sensitivity. *International Archives of Occupational and Environmental Health, 90*(2), 189-196. doi:10.1007/s00420-016-1185-8

Cannata A., De Luca, C., Andolina,G., Caccamo, D., Curro, M., Ferlazzo, N., Ientile, R., Alibrandi, A., and Korkina, L (2021) Influence of the SOD2 A16V gene polymorphism on alterations of redox markers and erythrocyte membrane fatty acid profiles in patients with multiple chemical sensitivity.
Biomedical Reports 15: 101, 2021 DOI: 10.3892/br.2021.1477

Japan

Azuma, K., Uchiyama, I., Takano, H., Tanigawa, M., Azuma, M., Bamba, I., & Yoshikawa, T. (2013). Changes in cerebral blood flow during olfactory stimulation in patients with multiple chemical sensitivity: a multihttps://pubmed.ncbi.nlm.nih.gov/24278291/channel near-infrared spectroscopic study. *PloS One, 8*(11), e80567. doi:10.1371/journal.pone.0080567

Azuma, K., Uchiyama, I., Katoh, T., Ogata, H., Arashidani, K., & Kunugita, N. (2015a). Prevalence and
Characteristics of Chemical Intolerance: A Japanese Population-Based Study. *Archives of Environmental*
& Occupational Health, 70(6), 341-353. doi:10.1080/19338244.2014.926855

Azuma, K., Uchiyama, I., Tanigawa, M., Bamba, I., Azuma, M., Takano, H., . . . Sakabe, K. (2015b). Assessment of cerebral blood flow in patients with multiple chemical sensitivity using near-infrared spectroscopy-recovery after olfactory stimulation: a case-control study. *Environmental Health and Preventive Medicine, 20*(3), 185-194. doi:10.1007/s12199-015-0448-4

Azuma, K., Uchiyama, I., Tanigawa, M., Bamba, I., Azuma, M., Takano, H., . . . Sakabe, K. (2016). Association of Odor Thresholds and Responses in Cerebral Blood Flow of the Prefrontal Area during Olfactory Stimulation in Patients with Multiple Chemical Sensitivity. *PloS One, 11*(12), e0168006.
doi:10.1371/journal.pone.0168006

Azuma, K., Uchiyama, I., & Kunugita, N. (2019). Factors affecting self-reported chemical intolerance: A fivehttps://pubmed.ncbi.nlm.nih.gov/30782347/year follow-up study in Japan. *Journal of Psychosomatic Research, 118*, 1-8.
doi:10.1016/j.jpsychores.2019.01.001

Cui, X., Lu, X., Hiura, M., Oda, M., Miyazaki, W., & Katoh, T. (2013). Evaluation of genetic polymorphisms in patients with multiple chemical sensitivity. *PloS One, 8*(8), e73708-e73708. doi:10.1371/journal.pone.0073708

Cui, X., Lu, X., Hiura, M., Oda, M., Hisada, A., Miyazaki, W., . . . Katoh, T. (2014). Prevalence and interannual changes in multiple chemical sensitivity in Japanese workers. *Environmental Health and Preventive Medicine, 19*(3), 215-219. doi:10.1007/s12199-014-0378-6

Cui, X., Lu, X., Hisada, A., Fujiwara, Y., & Katoh, T. (2015). The correlation between mental health and multiple chemical sensitivity: a survey study in Japanese workers. *Environmental Health and Preventive Medicine, 20*(2), 123-129. doi:10.1007/s12199-014-0434-2

Fujimori, S., Hiura, M., Yi, C. X., Xi, L., & Katoh, T. (2012). Factors in genetic susceptibility in a chemical sensitive population using QEESI. *Environmental Health and Preventive Medicine, 17*(5), 357-363.
doi:10.1007/s12199-011-0260-8

Hasegawa, M., Ohtomo, M., Mizuki, M., & Akiyama, K. (2009). [Diagnosis of multiple chemical sensitivity by chemical compounds exposure tests]. *Arerugi, 58*(2), 112-118.

Hojo, S. (2002). A questionnaire survey of multiple chemical sensitivity in Japan by using QEESI. *NeuroOphthalmology Japan, 19*(2), 169-175.

Hojo, S., Kumano, H., Yoshino, H., Kakuta, K., & Ishikawa, S. (2003). Application of Quick Environment Exposure
Sensitivity Inventory (QEESI©) for Japanese population: study of reliability and validity of the
questionnaire. *Toxicology and Industrial Health, 19*(2-6), 41-49. doi:10.1191/0748233703th180oa

Hojo, S., Yoshino, H., Kumano, H., Kakuta, K., Miyata, M., Sakabe, K., . . . Ishikawa, S. (2005). Use of QEESI© questionnaire for a screening study in Japan. *Toxicology and Industrial Health, 21*(3-4), 113-124. doi:10.1191/0748233705th219oa

Hojo, S., Ishikawa, S., Kumano, H., Miyata, M., & Sakabe, K. (2008). Clinical characteristics of physicianhttps://pubmed.ncbi.nlm.nih.gov/18155642/diagnosed patients with multiple chemical sensitivity in Japan. *International Journal of Hygiene and Environmental Health, 211*(5-6), 682-689. doi:10.1016/j.ijheh.2007.09.007

Hojo, S., Sakabe, K., Ishikawa, S., Miyata, M., & Kumano, H. (2009). Evaluation of subjective symptoms of Japanese patients with multiple chemical sensitivity using QEESI©. *Environmental Health and Preventive Medicine, 14*, 267-275.

Hojo, S., Mizukoshi, A., Azuma, K., Okumura, J., Ishikawa, S., Miyata, M., . . . Sakabe, K. (2018). Survey on changes in subjective symptoms, onset/trigger factors, allergic diseases, and chemical exposures in the past decade of Japanese patients with multiple chemical sensitivity. *International Journal of Hygiene and Environmental Health, 221*(8), 1085-1096. doi:10.1016/j.ijheh.2018.08.001

Hojo, S., Mizukoshi, A., Azuma, K., Okumura, J., Mizuki, M., & Miyata, M. (2019). New criteria for multiple chemical sensitivity based on the Quick Environmental Exposure and Sensitivity

Inventory developed in response to rapid changes in ongoing chemical exposures among Japanese. *PloS One, 14*(4), e0215144e0215144. doi:10.1371/journal.pone.0215144

Ishibashi, M., Tonori, H., Miki, T., Miyajima, E., Kudo, Y., Tsunoda, M., Sakabe, K., & Aizawa, Y. (2007).
Classification of patients complaining of sick house syndrome and/or multiple chemical sensitivity.
Tohoku Journal of Experimental Medicine, 211(3), 223-233. https://doi.org/10.1620/tjem.211.223

Lu, X., Hisada, A., Anai, A., Nakashita, C., Masuda, S., Fujiwara, Y., . . . Katoh, T. (2020). Study of the Correlation
Between Multiple Chemical Sensitivity and Personality Using the Quick Environmental Exposure
Sensitivity Inventory Questionnaire and the Temperament and Character Inventory. *Journal of*
Occupational and Environmental Medicine, 62(7), e348-e354. doi:10.1097/jom.0000000000001899

Manabe, R., Kunugita, N., Katoh, T., Kuroda, Y., Akiyama, Y., Yamano, Y., . . . Arashidani, K. (2008). [Questionnaire survey of workers in specific buildings regarding multiple chemical sensitivity]. *Nihon Eiseigaku Zasshi. Japanese Journal of Hygiene, 63*(4), 717-723. doi:10.1265/jjh.63.717

Mizuki, M. (2004) Effect of environmental factors in patients with multiple chemical sensitivity. *IRYO-Japanese Journal of National Medical Services, 58*(7), 399-407.

Mizuki, M. (2015) Intractable factors in patients with chemical sensitivity. *IRYO-Japanese Journal of National Medical Services, 69*(3), 117-126.

Mizukoshi, A., Kumagai, K., Yamamoto, N., Noguchi, M., Yoshiuchi, K., Kumano, H., . . . Yanagisawa, Y. (2015). In-situ Real-Time Monitoring of Volatile Organic Compound Exposure and Heart Rate Variability for
Patients with Multiple Chemical Sensitivity. *International Journal of Environmental Research and Public Health, 12*(10), 12446-12465. doi:10.3390/ijerph121012446

Nakaoka, H., Suzuki, N., Nakayama, Y., Takaya, K., Todaka, E., and Mori, C. (2018). A preliminary study to investigate the relationships between indoor environment and its effect on physical and mental health. WIT Transactions on Ecology and the Environment, *230*, 567-576.

Ohsawa, M., Takayama, S., Kikuchi, A., Arita, R., Saito, N., Hojo, S., . . . Yaegashi, N. (2020). Two Cases of Multiple Chemical Sensitivity Successfully Treated With Kampo Medicine. *Alternative Therapies in Health and Medicine.*

Suzuki, N., Nakaoka, H., Nakayama, Y., Tsumura, K., Takaguchi, K., Takaya, K., . . . Mori, C. (2020). Association between sum of volatile organic compounds and occurrence of building-related symptoms in humans: A study in real full-scale laboratory houses. *Science of the Total Environment, 750*, 141635.
doi:10.1016/j.scitotenv.2020.141635

Watai, K., Fukutomi, Y., Hayashi, H., Kamide, Y., Sekiya, K., & Taniguchi, M. (2018). Epidemiological association between multiple chemical sensitivity and birth by caesarean section: a nationwide case-control study. *Environmental Health, 17*(1), 89. doi:10.1186/s12940-018-0438-2

Yoshino, H., Amano, K., Matsumoto, M., Netsu, K., Ikeda, K., Nozaki, A., . . . Ishikawa, S. (2004). Long-Termed Field Survey of Indoor Air Quality and Health Hazards in Sick House. *Journal of Asian Architecture and Building Engineering, 3*(2), 297-303. doi:10.3130/jaabe.3.297

Saudi Arabia

Khalil, A. I., Almutairi, M. S., & Ahmed, M. E. (2020). Assessing risk factors of Autism Spectrum Disorders (ASD) and Attention Deficit Hyperactivity Disorder (ADHD) among Saudi Mothers: A retrospective study [Article]. *Clinical Schizophrenia and Related Psychoses*, *14*(1), Article 092320. https://doi.org/10.3371/CSRP.IASM.092320

South Korea

Heo, Y., Kim, S. H., Lee, S. K., & Kim, H. A. (2017). Factors Contributing to the Self-Reported Prevalence of Multiple Chemical Sensitivity in Public Facility Workers and the General Population of Korea. *J uoeh, 39*(4), 249-258. doi:10.7888/juoeh.39.249

Jeon, B. H. L., S.H.; Kim H.A. (2012). A Validation of the Korean Version of QEESI(c) (The Quick Environmental Exposure and Sensitivity Inventory). *Korean Journal of Occupational and Environmental Medicine., 24*(1), 96-114.

Jeong, I., Kim, I., Park, H. J., Roh, J., Park, J. W., & Lee, J. H. (2014). Allergic diseases and multiple chemical sensitivity in Korean adults. *Allergy, Asthma & Immunology Research, 6*(5), 409-414. doi:10.4168/aair.2014.6.5.409

Yun, M. J., Kang, D. M., Lee, K. H., Kim, Y. K., & Kim, J. E. (2013). Multiple chemical sensitivity caused by exposure to ignition coal fumes: a case report. *Ann Occup Environ Med, 25*(1), 32. doi:10.1186/20524374-25-32

Byung Woo Kwon, Woo Yong Bae, Nam Yoon Jung, Chang Bae Lee, Daeyeon Kim (2019). Chemical Intolerance in Patients With Nasal Inflammatory Disorders. Archives of Clinical and Biomedical Research Vol. 3 No. 4 - August 2019. [ISSN 2572-5017] 257; Arch Clin Biomed Res 2019; 3 (4): 257-271

Spain

Aguilar-Aguilar, E., Marcos-Pasero, H., de la Iglesia, R., Espinosa-Salinas, I., Ramírez de Molina, A., Reglero, G., & Loria-Kohen, V. (2018). Characteristics and determinants of dietary intake and

physical activity in a group of patients with multiple chemical sensitivity. *Endocrinol Diabetes Nutr*, *65*(10), 564-570. doi:10.1016/j.endinu.2018.07.009

Alobid, I., Nogué, S., Izquierdo-Dominguez, A., Centellas, S., Bernal-Sprekelsen, M., & Mullol, J. (2014). Multiple chemical sensitivity worsens quality of life and cognitive and sensorial features of sense of smell. *European Archives of Oto-Rhino-Laryngology, 271*(12), 3203-3208. doi:10.1007/s00405-0143015-5

Fernandez-Solà, J. (2005). Chronic fatigue syndrome and fibromyalgia in patients affected with multiple chemical sensitivity. Med Clin (Barc); 124: 451-453.

García-Sierra, R., & Álvarez-Moleiro, M. (2014). Evaluation of suffering in individuals with multiple chemical sensitivity. *Clínica y Salud, 25*(2), 95-103. doi:https://doi.org/10.1016/j.clysa.2014.06.006

Lago Blanco, E., Puiguriguer Ferrando, J., Rodríguez Enríquez, M., Agüero Gento, L., Salvà Coll, J., & Pizà Portell, M. R. (2016). Multiple chemical sensitivity: Clinical evaluation of the severity and psychopathological profile. *Medicina Clínica, 146*(3), 108-111. https://doi.org/10.1016/j.medcli.2015.09.016

Mena, G., Sequera, V. G., Nogué-Xarau, S., Ríos, J., Bertran, M. J., & Trilla, A. (2013). [Translation and crosshttps://pubmed.ncbi.nlm.nih.gov/23347502/cultural adaptation of the Quick Environmental Exposure and Sensitivity Inventory for use in the Spanish population]. *Medicina Clínica, 140*(7), 302-304. doi:10.1016/j.medcli.2012.10.024

Nogué, S., Fernández-Solá, J., Rovira, E., Montori, E., Fernández-Huerta, J. M., & Munné, P. (2007). [Multiple chemical sensitivity: study of 52 cases]. *Medicina Clínica, 129*(3), 96-98; quiz 99. doi:10.1157/13107370

Paredes Rizo, M. (2018). Multiple Chemical Sensitivity: analysis of a case registered in a reference Hospital. *Medicine and Occupational Safety*, *64* (251), 217-240.

Pérez-Crespo, J., Lobato-Cañón, R., & Solanes-Puchol, Á. (2018). Multiple Chemical Sensitivity in Chemical Laboratory Workers. *Safety and health at work, 9*(4), 473-478. doi:10.1016/j.shaw.2018.03.001

Loria-KohenV, Marcos-PaseroH, de la IglesiaR, Aguilar-Aguilar E, Espinosa-Salinas I, Herranz J, et al. Sensibilidad química múltiple: caracterización genotípica, estado nutricional y calidad de vida de 52 pacientes. Med Clin (Barc). 2017;149:141–146.

Sweden

Andersson, M. J., Andersson, L., Bende, M., Millqvist, E., & Nordin, S. (2009). The idiopathic environmental intolerance symptom inventory: development, evaluation, and application. *Journal of Occupational and Environmental Medicine, 51*(7), 838-847. doi:10.1097/JOM.0b013e3181a7f021

Nordin, S., & Andersson, L. (2010). Evaluation of a Swedish version of the Quick Environmental Exposure and Sensitivity Inventory. *International Archives of Occupational and Environmental Health, 83*(1), 95-104.
doi:10.1007/s00420-009-0427-4

United States

Gould Peek, G., & Wallace Lyon, M. (2014). Housing and Health Relationship: Multiple Chemical Sensitivity (MCS). *Housing and Society, 41*(1), 31-52. doi:10.1080/08882746.2014.11430620

Heilbrun, L. P., Palmer, R. F., Jaen, C. R., Svoboda, M. D., Perkins, J., & Miller, C. S. (2015). Maternal Chemical and Drug Intolerances: Potential Risk Factors for Autism and Attention Deficit Hyperactivity Disorder (ADHD). *Journal of the American Board of Family Medicine, 28*(4), 461-470.
doi:10.3122/jabfm.2015.04.140192

Katerndahl, D. A., Bell, I. R., Palmer, R. F., & Miller, C. S. (2012). Chemical intolerance in primary care settings:
prevalence, comorbidity, and outcomes. *Annals of Family Medicine, 10*(4), 357-365.
doi:10.1370/afm.1346

Miller, C. S., & Prihoda, T. J. (1999a). The Environmental Exposure and Sensitivity Inventory (EESI): a standardized approach for measuring chemical intolerances for research and clinical applications.
Toxicology and Industrial Health, 15(3-4), 370-385. doi:10.1177/074823379901500311

Miller, C. S., & Prihoda, T. J. (1999b). A controlled comparison of symptoms and chemical intolerances reported by Gulf War veterans, implant recipients and persons with multiple chemical sensitivity.
Toxicology and Industrial Health, 15(3-4), 386-397. doi:10.1177/074823379901500312

Palmer, R. F., Jaén, C. R., Perales, R. B., Rincon, R., Forster, J. N., & Miller, C. S. (2020). Three questions for identifying chemically intolerant individuals in clinical and epidemiological populations: The Brief Environmental Exposure and Sensitivity Inventory (BREESI). *PloS One, 15*(9), e0238296.
doi:10.1371/journal.pone.0238296

Palmer, R. F., Walker, T., Kattari, D., Rincon, R., Perales, R. B., Rincon, R., Jaen, C. R., Grimes, C., Sundbland, D. R., & Miller, C. S. (2020). Validation of a Brief Screening Instrument for Chemical Intolerance in a Large U.S. National Sample. Int. J. Environ. Res. Public Health 2021, 18, 8714. https://doi.org/10.3390/ijerph18168714

Miller, C.S., Palmer, R., Dempsey, T., Ashford, N. A., Afrin L.B. (2021) Mast cell activation may explain many cases of chemical intolerance Environmental Sciences Europe (2021) 33:129 https://doi.org/10.1186/s12302-021-00570-3

Masri, S., Miller, C.S., Palmer, R., Ashford, N. Toxicant-Induced Loss of Tolerance for chemicals, foods, and drugs: assessing patterns of exposure behind a global phenomenon. Environ Sci Eur (2021) 33:65 https://doi.org/10.1186/s12302-021-00504-z

Uruguay

De Ben, S., Sponton, F. Chaves, E., Medina, F., Tomasina, F. (2014.) Multiple chemical sensitivity: a challenge for occupational health. Rev Med Urug, 30: 123-127.
